# Research on safety and compliance of imported microbial inoculants using high-throughput sequencing

**DOI:** 10.3389/fmed.2022.963988

**Published:** 2022-09-21

**Authors:** Lin Dong, Zilong Zhang, Biyun Zhu, Shenwei Li, Yan He, Yating Lou, Ping Li, Huajun Zheng, Zhengan Tian, Xia Ma

**Affiliations:** ^1^School of Perfume and Aroma Technology, Shanghai Institute of Technology, Shanghai, China; ^2^Shanghai International Travel Healthcare Center, Shanghai, China; ^3^Shanghai-MOST Key Laboratory of Health and Disease Genomics, Chinese National Human Genome Center at Shanghai, Shanghai Institute for Biomedical and Pharmaceutical Technologies, Shanghai, China

**Keywords:** microbial inoculants, high-throughput sequencing, biosafety, non-targeted detection, qPCR

## Abstract

Microbial inoculants are widely used in wastewater treatment, soil remediation, and biological control. Safety and compliance for active constituents are considered to be the most important measures of imported microbial inoculants. Microbial inoculants composition was commonly identified by phenotypic culture, which is time-consuming and labor intense with occasionally false negative results provided, and can only be tested for specific species. High-throughput sequencing (HTS), known for its non-targeted detection of unknown species composition in samples, is suitable for composition consistency identification and biosafety analysis of imported microbial inoculants. In this study, the application of HTS for microflora distribution and resistance gene was verified in microbial inoculants for environmental protection and then applicated in imported microbial inoculants. Both Illumina- and Nanopore-based HTS methods identified the same dominant bacterial species successfully in the imported microbial inoculants. The main component of bacterial species was *Bacillus subtilis, Bacillus amyloliquefaciens, Bacillus licheniformis*, and *Enterococcus faecium*, and further confirmed with traditional methods. The antibiotic resistance genes *Bacillus subtilis mprF, bcrA, blt, lmrB, rphB, tet(L), tmrB, vmlR, ykkC, and ykkD* were detected in all samples. Our results indicated that HTS processes the application potential to identify the active ingredients of microbial inoculants. Therefore, rapid and accurate identification of the microbial compositions in microbial formulation products is of high importance for port biosafety supervision.

## Introduction

Industrialization and urbanization have led to environmental deterioration, soil erosion, water pollution, biodiversity decline, and desertification (1, 2). Prevention and control of environmental pollution bear heavy responsibilities through a long struggle (3). Microbial inoculants are widely used in wastewater treatment, soil remediation, and biological control for their low cost, minimum toxicity, reduced residues, simplicity, and high efficiency (4, 5). Ecological adaptation and microbial community-preserving capacity are important criteria when assessing the suitability of bio-inoculants for commercial development (6). However, microbial inoculants vary in quality. Transboundary spread of non-dominant component bacterial species may lead to microbes invading, which will pose potential threats to the environment and human health (7). Therefore, the accurate identification of microbial components is of high importance in evaluating the quality and safety of microbial inoculants. Only the samples that have passed the detection at the customs and were judged to be qualified according to the relevant standards are allowed to enter the country.

Early recognition of hazardous biological materials is essential to both biodefense and biosafety strategies (8). Detection and identification of these substances, which lack a rapid and accurate detection method, are the major challenges for customs laboratories. At present, the identification of imported microbial inoculants for environmental protection in China is in accordance with the Import and Export Industry Standard “Inspection Methods for Entry Microbial Inoculants for Environmental Protection” (9). This standard is mainly based on traditional microbial detection methods, such as morphological observation, biochemical reaction identification, polymerase chain reaction (PCR) identification, and fluorescent quantitative PCR (qPCR) identification (10). However, several challenges are facing in its practical applications: (i) Although the common microbial species in microbial inoculants have been covered (17 target bacteria species), species that are not in the catalog cannot be identified and certified; (ii) The specificity and effectiveness of PCR primers for target bacteria cannot be guaranteed, which may result in false negative or false positive results; (iii) culture-dependent bacterial identification relies on a pure culture of the strains, which are labor and time consuming (11). Therefore, the accuracy of current methods is affected by the complexity of microbial samples and unavoidable operational errors.

High-throughput sequencing (HTS) is now widely used in the identification of pathogenic bacteria. It can detect unknown complex samples without plate culture directly with high accuracy and allowed for hundreds of microbial communities to be simultaneously assayed (12–14). Next-generation sequencing (NGS) is the most popular HTS technology and has been widely used on soil, water, and air samples in microbial composition identification (15). Advances in low-cost, high-throughput DNA sequencing technologies have enabled the studies of the composition of microbial communities at unprecedented throughput levels (16). Illumina is locked in the mainstream market of NGS sequencing. Meanwhile, long-read sequencing devices from Oxford Nanopore Technologies (ONT) offer an alternative with several advantages (17). In comparison to NGS, nanopore sequencers are higher cost but faster when targeting microbes with small genomes. Nanopore sequencing has proven to be a fast and convenient method for studying pathogens in water bodies (18).

In this study, we updated HTS-based methods for safety and consistency of microbial inoculants testing. Four commercial microbial inoculants were used for method suitability evaluation. Illumina sequencing and Nanopore sequencing were compared with industry standard methods (SN/T 4624-2016) (9) for practical testing with imported samples. Our results indicated that HTS processes high feasibility and accuracy for the identification of bacterial components in microbial inoculants for environmental protection.

## Materials and methods

### Sample collection

Four commercial microbial inoculants were purchased from different manufacturers, numbered as Y-1, Y-2, Y-3, and Y-4. Shanghai Customs, China provided the inbound microbial inoculants for environmental protection in yellow powder form.

### DNA extraction

Samples were extracted using Magnetic Soil and Stool DNA Kit (Tiangen, Shanghai, China) (19). Briefly, samples (0.5 g) were added with 500 μl Buffer SA, 100 μl Buffer SC, and 0.25 g grinding bead. The supernatant was removed by centrifugation after 15 min of vortex oscillation. Buffer SH was added to pellet and mixed by vertexing. The supernatant was collected after centrifugation at 4^°^C for 10 min. Buffer GFA was added to the supernatant and mixed upside down, then magnetic bead suspension G was added and shacked for 5 min. It was then placed on a magnetic rack to be absorbed completely. Deproteinizing solution RD was added and washed with washing buffer PWD twice. Finally, target DNA was dissolved with 100 μl Buffer TB (20, 21). DNA was quantified using Qubit (Invitrogen, Carlsbad, CA, USA) (22).

### Illumina sequencing

Extracted DNA (100 ng) was fragmented into 300 bp randomly using Covaris S2 sonicator (Covaris, Woburn, MA, USA). The library was constructed using NEXTflex DNA sequencing kit (BioAim Scientific, Toronto, ON, Canada) and purified with AMX pure magnetic beads (Beckman, CA, USA). Quality control was conducted using Onedrop quantification, 2% agarose gel electrophoresis assay, and a high sensitivity DNA chip assay (23). Paired-end sequencing was performed on the Illumina Hiseq 2500 with 10 ng library. The metagenomic data have been deposited in the NCBI Sequence Read Archive under accession numbers PRJNA853488, SRR19880285-SRR19880288, and SRR20082797, respectively.

FASTX-Toolkit^1^ was used to filter the original data of Illumina sequencing (trim SLIDINGWINDOW:4:20 MINLEN:50) and obtain valid data by removing the low-quality sequences. Sequences passed quality control were compared with the NCBI nt database for BLAST homology with the parameter *e*-value < 1e-20 (24, 25). The results were then imported into MEGAN software v6.21.12 (26) to assign each sequence. Metagenomic reads were assembled into contigs using MegaHIT v1.2.9 (27) individually for each sample, followed by an open reading frame prediction using Prodigal v2.6.2 (28). For the annotation of antibiotic resistance genes, the Comprehensive Antibiotic Resistance Database (CARD)^2^ release 3.2.3 (22/06/14) (29), was used as the reference functional classification, and the reads were annotated using the best hit to this CARD database. The results from this annotation were manually curated.

### Oxford nanopore sequencing

The ligation sequencing kit (SQK-LSK109, Oxford Nanopore Technologies, Oxford, UK) was used and then real-time sequencing technique, MinION (Oxford Nanopore Technologies, Oxford, UK) was used (30). Briefly, DNA was extracted with Magnetic Soil and Stool DNA Kit (Tiangen, China). The concentration of the extracted DNA (∼1 μg) was measured with Qubit fluorometer (Thermo Fischer Scientific, Waltham, MA, USA). For fragmented DNA repair and end-repaired DNA, NEBNext FFPE repair Mix and NEBNext end repair/dA-tailing Module (New England BioLabs Inc., Ipswich, MA, USA) was used, respectively. After DNA purification with AMPure XP beads (Beckman Coulter, Brea, CA, USA), the sample was loaded on the Minion Flow Cell R9.4 (Oxford Nanopore Technologies, Oxford, UK). Sequencing protocol was applied using the nanopore sequencing software, MinKNOW (v1.10.23, 2017, Oxford Nanopore Technologies, Oxford, UK), in order to collect electronic signal data. After basecalling of long reads with Oxford Nanopore software GUPPY 3.0.3 (to convert fast5 files in fasta format), each output file from the nanopore sequencing was BLAST searched against the “nucleotide collection (nt)” database from NCBI. Complete BLAST outputs for each sample were imported into MEGAN v6.21.12 using the default parameters.

### Quantitative PCR verification

The safety of environmental microbial strains was measured according to the “Guidelines for Environmental Safety Assessment of Microbial Species for Environmental Protection” of the General Administration of Quality Supervision, Inspection and Quarantine (31). Human-borne pathogens (32) and zoonotic pathogens (33) are classified according to the Regulations on Biosafety Management of Pathogenic Microorganism Laboratories (34). *Bifidobacterium*, denitrifying bacteria, *Saccharomyces*, and photosynthetic bacteria were identified, respectively, using commercial Bifidobacterium qPCR Kit (Yaji Biotech, Shanghai, China), denitrifying bacteria qPCR Kit (Yuxiu Biotech, Shanghai, China), Blastocystis spp. qPCR Kit (Lianmai Bioengineering, Shanghai, China) and SYBR Green I Kit (Sobao Biotech, Shanghai, China). The qPCR system was conducted in a final volume of 20 μl containing Premix Taq (TaKaRa, Otsu, Japan) (10 μl), 10 mM primer (0.2 μl each), DNA templates (40 ng), and ddH_2_O (35). Identification of qPCR was performed on ViiA7 Realtime PCR (ABI, New York, USA). The primer sequences and reaction conditions were as Supplementary Table 1.

## Results

### Establishment and validation of high-throughput sequencing methods

Y-1, Y-2, Y-3, and Y-4 microbial products were sequenced using the Illumina Hiseq 2500 platform, and obtained 1967.97, 1498.04, 1710.26, and 1632.48 Mb of valid data, respectively. The percentage of available data of Q20 and Q30 was more than 96 and 91%, respectively, indicating the reliable raw data could be used for species annotation (Supplementary Table 2).

**FIGURE 1:**
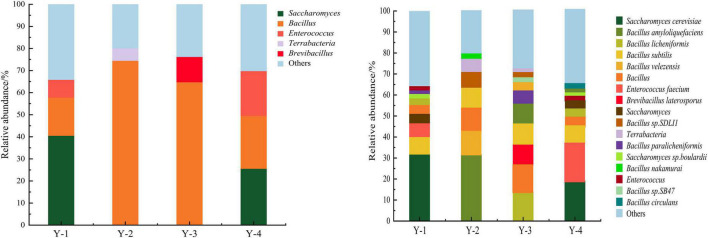
Relative abundance of microorganisms at genus level **(left)** and species level **(right)** of four commercial microbial inoculants.

The microbial composition of four commercial microbial inoculants was analyzed at the genus level (Figure 1). According to the relative abundance, microorganisms were divided into dominant genera (relative abundance ≥ 1.0%) and minor genera (relative abundance < 1.0%) (36). In Y-1, *Saccharomyces*, *Bacillus*, and *Enterococcus* were dominant genera, with relative abundances of 40.53, 17.27, and 8.09%, respectively. In Y-2, *Bacillus* and *Terrabacteria* were dominant genera, with relative abundances of 74.49 and 5.58%. In Y-3, *Bacillus* and *Brevibacillus* were dominant genera, with relative abundances of 64.77 and 11.48%. *Saccharomyces*, *Bacillus*, and *Enterococcus* were the dominant genera in Y-4, with abundances of 25.06, 23.90, and 20.32%, respectively.

The microbial composition of four commercial microbial inoculants was then analyzed at the species level (Figure 1). At the species level, 17 dominant species were identified. Nine dominant species were obtained from Y-1 samples, such as *Saccharomyces cerevisiae* (32.09%), *Bacillus subtilis* (7.99%), *Enterococcus faecium* (6.36%), *Saccharomyces* (4.74%), *Bacillus* (3.66%), *Bacillus licheniformis* (2.68%), *Saccharomyces* sp. *Boulardii* (2.46%), *Bacillus paralicheniformis* (1.31%), and *Enterococcus* (1.19%). A total of seven dominant species such as *Bacillus amyloliquefaciens* (31.79%), *Bacillus velezensis* (11.82%), *Bacillus* (10.58%), *Bacillus subtilis* (9.91%), *Bacillus* sp. *SDLI1* (6.26%), *Terrabacteria* (5.58%), and *Bacillus nakamurai* (2.66%) were identified in Y-2. Ten dominant species were obtained from Y-3 samples, namely, *Bacillus licheniformis* (13.53%), *Bacillus* (12.04%), *Bacillus subtilis* (10.99%), *Bacillus amyloliquefaciens* (10.28%), *Brevibacillus laterosporus* (10.07%), *Bacillus paralicheniformis* (6.93%), *Bacillus velezensis* (3.32%), *Bacillus* sp. *SB47* (2.00%), *Bacillus* sp. *SDLI1* (1.81%), and *Terrabacteria* (1.69%). And for Y-4 samples, 10 dominant species such as *Saccharomyces cerevisiae* (19.19%), *Enterococcus faecium* (16.04%), *Bacillus subtilis* (9.71%), *Bacillus* (6.59%), *Bacillus licheniformis* (3.87%), *Saccharomyces* (3.19%), *Enterococcus* (2.44%), *Saccharomyces* sp. *Boulardii* (1.93%), *Bacillus amyloliquefaciens* (1.03%), and *Bacillus circulans* (1.02%) were identified. No pathogenic bacteria were identified in tested four commercial microbial inoculants, indicating a safe composition of microbial strains for human health.

### Comparison of the identified strains with product description

The microbial compositions of Illumina sequencing were compared with product description. Strains declared in Y-1 and Y-3 products description were identified, while some strains declared in Y-2 and Y-4 samples could not be found. Namely, *Bifidobacterium*, denitrifying bacteria, *Saccharomyces*, and photosynthetic bacteria were not found in Y-2 samples, and photosynthetic bacteria and denitrifying bacteria were not found in Y-4 samples (Table 1).

**TABLE 1:** Comparison of the identified strains with product description.

Sample name	Labeled species	Identified	Abundance/%
Y-1	*Bacillus*	+	3.66
	*Saccharomyces*	+	4.74
	*Enterococcus*	+	1.19
Y-2	*Bacillus*	+	10.58
	*Bacillus subtilis*	+	9.91
	*Terrabacteria*	+	5.58
	*Bifidobacterium*	–	0
	Denitrifying bacteria	–	0
	*Enterococcus*	+	0.10
	*Saccharomyces*	–	0
	Photosynthetic bacteria	–	0
Y-3	*Bacillus subtilis*	+	10.99
	*Bacillus licheniformis*	+	13.53
	*Bacillus amyloliquefaciens*	+	10.28
	*Brevibacillus laterosporus*	+	10.07
Y-4	*Saccharomyces*	+	3.19
	*Enterococcus*	+	2.44
	Photosynthetic bacteria	–	0
	Denitrifying bacteria	–	0
	*Bacillus*	+	6.59

“ + ” indicates identified in related sample. “–” indicates not identified in related sample.

Missing strains but declared in products description were further identified with qPCR to verify the method accuracy of HTS in bacteria identification of microbial inoculums. However, none of the missing strains in HTS method was tested positive by qPCR (Supplementary Figure 1).

### Drug resistance gene analysis

Drug resistance genes could also be identified through HTS. The drug resistance gene numbers of four samples were different in the CARD, which are 31, 13, 42, and 39 genes, respectively. The specific numbers and characteristics of genes are presented in the Supplementary Table 3. *Bacillus subtilis mprF, bcrA, blt, lmrB, rphB, tet(L), tmrB, vmlR, ykkC*, and *ykkD* were detected in all samples. Some genes were repetitively detected in the same sample, including *Bacillus subtilis mprF, bcrA, blt, FosM1, rphB, vmlR, ykkC*, and *ykkD.* Resistance mechanisms were assigned to five categories as follows: antibiotic efflux, antibiotic inactivation, antibiotic target alteration, antibiotic target protection, and reduced permeability to antibiotics.

### Lower sequencing depth limit of quantification

**FIGURE 2:**
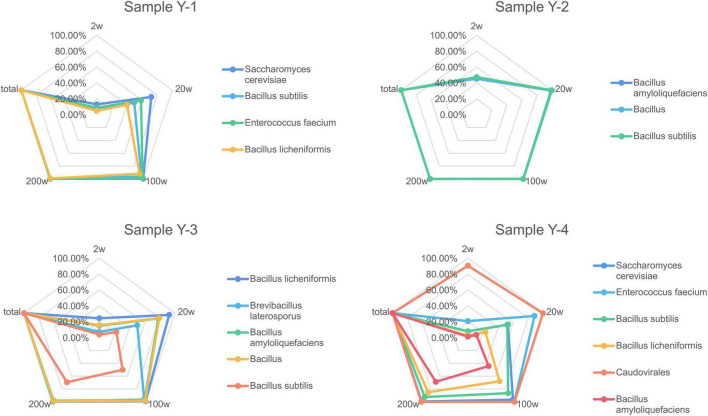
Completeness of recovered genome fraction calculated by comparing the assembled draft genomes for each species depending on sequencing depth. Assemblies were generated after drawn at random with 2, 20, 100, and 200 w reads.

In order to verify the usage of different sequencing depths has consequences on the completeness of draft genome assemblies from the environmental protection inoculants, metagenomics assemblies were generated at different read depths. The recovered genome fraction calculated by comparing the assembled draft genomes for each species (Figure 2) shows few differences between sequencing depth for some species, especially with samples containing more bacterial species. A sequencing depth of 200 w, which is similar to the actual sequencing depth for the metagenomics dataset, generated approximately the same dissimilarity profile as the real data. When comparing the total recovered genomes from all species of the environmental protection inoculants at different read depths, it is difficult to detect low-abundance species when the sample has complex species. Detection sensitivity dramatically increased with increasing sequencing depth and achieved 95% coverage (14/18, 77.78%) when the sequencing depth was greater than 200 w.

### High-throughput sequencing methods comparison based on imported microbial inoculants

**FIGURE 3:**
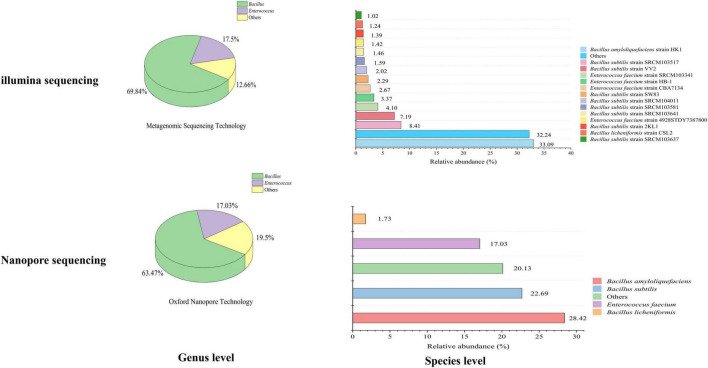
Relative abundance of microorganisms in inbound environmental protection microbial inoculants using illumina and nanopore sequencing. Genus level **(left)** and species level **(right)**.

Based on Illumina methods, the microbial composition of imported environmental protection inoculants was identified at genus and species level. Two dominant genera, *Bacillus* and *Enterococcus*, were identified with the relative abundance of 69.84 and 17.50% on the genus level (Figure 3). Four dominant species, *Bacillus amyloliquefaciens*, *Bacillus subtilis*, *Enterococcus faecium* and *Bacillus licheniformis* were verified on the species level with relative abundance of 33.09, 25.37, 11.16, and 1.24%. The relative abundance of the dominant species were *Bacillus amyloliquefaciens* strain HK1 (33.09%), *Bacillus subtilis* strain SRCM103517 (8.41%), *Bacillus subtilis* strain VV2 (7.19%), *Bacillus subtilis* strain SW83 (2.29%), *Bacillus subtilis* strain SRCM104011 (2.02%), *Bacillus subtilis* strain SRCM103581 (1.59%), *Bacillus subtilis* strain SRCM103641 (1.46%), *Bacillus subtilis* strain 2KL1 (1.39%), *Bacillus subtilis* strain SRCM103637 (1.02%), *Enterococcus faecium* strain SRCM103341 (4.10%), *Enterococcus faecium* strain HB-1 (3.37%), *Enterococcus faecium* strain CBA7134 (2.27%), *Enterococcus faecium* strain 4928STDY7387800 (1.42%), and *Bacillus licheniformis* strain CSL2 (1.24%).

While for Nanopore-based HTS methods, the microbial composition of the imported environmental protection inoculants was also analyzed at the genus and species level, respectively. Two dominant genera, *Bacillus* and *Enterococcus*, with relative abundances of 63.47 and 17.03% at the genus level were identified (Figure 3). The four dominant species observed were *Bacillus amyloliquefaciens* (28.42%), *Bacillus subtilis* (22.69%), *Enterococcus faecium* (17.03%), and *Bacillus licheniformis* (1.73%) in species-level analysis, which was consistent with Illumina-based methods mentioned above. None pathogenic bacteria were detected in the imported microbial inoculants through both sequencing methods.

## Discussion

In this study, HTS methods for microbial composition were established and verified with four commercial microbial inoculants. It was further allied on imported microbial inoculants. Among these five tested microbial inoculants, four bacterial genera were identified which were common genera in environmental protection microbial inoculant products and play important roles in environmental and biological control. Oliveira et al. found that *Saccharomyces* biosorption is a low-cost and effective method to remove Cd^2+^ from polluted water (37). Probiotic *Bacillus* has been demonstrated to process the ability to improving feed efficiency, stress response, immune response, and diseasing resistance of fish in sustainable aquaculture (38).

To evaluate HTS methods updated in this study, the traditional method (SN/T 4624-2016) was selected as a standard control for imported microbial inoculants identification. The traditional methods and HTS methods for inbound microbial inoculants were compared in terms of detection results and time-consumption (Table 2). The traditional method for the identification of microorganism was mainly based on agar plate culture, and was relatively time-consuming. Only specified target pathogens listed in the standard could be identified for their existence. Moreover, for the culture-based traditional method, it was complicated to identify the non-dominant strains under the same cultural conditions. HTS methods can identify bacterial species with a relative abundance of less than 1.0%. By comparing with the list of pathogenic bacteria, the microbial safety of microbial inoculants can be effectively evaluated by HTS, which are less time-consuming, higher-throughput, and non-targeted. Besides, a rich variety of *Bacillus* species was found in tested microbial inoculants. Due to the high sequence similarity (98.1–99.8%) and similar phenotypic differentiation (39), *Bacillus* strains are difficult and complex to identify by traditional methods. HTS method can solve this challenge and reduce the problems in identifying the species with complex microbial compositions.

**TABLE 2:** Comparison of traditional methods and sequencing methods for imported microbial inoculants.

Method	Traditional detection methods		Genetic testing methods	
	Laboratory routine microbiological methods	SN/T 4624-2016	Illumina sequencing technology	Nanopore sequencing technology
Principle	Based on agar plate culture, microorganisms are identified by analyzing morphological characteristics, physiological and biochemical reaction characteristics, PCR reaction and fluorescent PCR reaction.		Sequencing by synthesis, bridge PCR, sequencing while synthesizing DNA molecules.	Nanopore-based single-molecule real-time sequencing.
Test results	*Bacillus subtilis*		*Bacillus subtilis* (25.35%)	*Bacillus subtilis* (22.69%)
	*Bacillus amyloliquefaciens*		*Bacillus amyloliquefaciens* (33.09%)	*Bacillus amyloliquefaciens* (28.42%)
	*Bacillus licheniformis*		*Bacillus licheniformis* (1.24%)	*Bacillus licheniformis* (1.73%)
	*Enterococcus faecalis*		*Enterococcus faecalis* (11.16%)	*Enterococcus faecalis* (17.03%)
Time-consuming for detection	≧2 weeks		≦5 days	

Higher demands for the customs department must be placed on monitoring the bio-invasion security of imported products. It has been demonstrated by our results that the compositions of the tested four microbial inoculants were not always consistent with the product description (Table 1). Some strains declared on the labels were neither found by traditional method nor by HTS method. While some strains were identified with absence on the label of product. These inconsistent ingredients highlighted the necessity and feasibility in updating detection methods to control the quality of microbial inoculants. The inconsistency between product description and identified strains reflects two key issues in the current microbial inoculant market: Firstly, some manufacturers label their product ingredient lists with non-existent bacterial ingredients to increase their sales. Secondly, some functional bacterial species were hidden intentionally to protect the product ingredient confidentiality. These practices violate relevant national laws and regulations, and may cause disturbance to the local ecological environment. Therefore, rapid and accurate identification of the microbial compositions in microbial formulation products have high importance for port biosafety supervision.

Two HTS methods such as Illumina-based and nanopore-based sequencing methods were evaluated by the same imported microbial inoculant sample. There were no pathogenic bacteria identified by either method. While, divergence in the relative abundance of strains between the two methods was observed, which could be influenced by sequencing principles and depth, instruments, bioinformatics software, and databases (40–42). In addition, for the ingredients identification, the Nanopore-based HTS method can only verify at species level while the Illumina-based HTS method can identify at the strain level. High sequencing depth is also critical to sensitivity, and as the cost of sequencing continues to decline, high-depth sequencing is becoming more common practice. Depending on the sequencing depth, the identification of relevant microbial products data analysis performed at different resolutions. Low depth of sequencing often introduces sequencing biases and reduces variant calling sensitivity. Due to different depths of sequencing, the deeper depth of the sequencing is, the more composite microbial species that will be annotated. The sequencing depth has restricted differential detection of less abundantly microbes. Thus, it is significant to find the best balance between the cost of sequencing and the depth of the sequencing.

Metagenomics has the potential to become a powerful tool in the field of pathogenic microbes’ detection, since it allows the detection, identification, and characterization of a broad range of pathogens in a single experiment without pre-cultivation within a couple of days. 16S rDNA sequencing did not only result in high deviations from the expected sample composition on genus and species level, but more importantly lacked the detection of several pathogenic species (43). HTS is more suitable for species detection, abundance estimation, genome assembly, drug resistance, and species characterization. Antibiotic resistance genes could be identified simultaneously by HTS. Antibiotic resistance genes carried by imported microbial inoculants could spread by horizontal gene transfer between different bacterial communities, leading to the widespread prevalence of drug resistance genes and the emergence of multidrug resistance (44, 45). Many reports have indicated that the resistance epidemiology is global and spreads through nations and across borders (46). Evaluation of resistant profiles and detection of antimicrobial-resistant genes of bacterial pathogens in the microbial inoculants is imperative to assess the probable risk of dissemination of resistant genes in the environment (47). The present study proved that HTS can be an effective approach in the safety and compliance of imported microbial inoculants.

By comparing with traditional methods, HTS methods process a larger detection spectrum and broad application prospects with high accuracy. Thus, HTS provides a new and effective approach to the safety and compliance of imported microbial inoculants for quarantine departments. Especially from the two aspects of product safety and compliance, it is necessary to conduct a comprehensive evaluation of the active ingredients of imported microbial inoculants.

## Data availability statement

The data presented in this study are deposited in the National Center for Biotechnology Information (NCBI), accession numbers: PRJNA 853488, SRR 19880285–SRR 19880288, and SRR 20082797, respectively. The data of this study has been published.

## Authors contributions

LD, ZZ, and BZ: conceptualization and writing—original draft. LD, ZZ, BZ, SL, YL, XM, ZT, and YH: investigation, methodology, data curation, and formal analysis. ZZ and ZT: funding acquisition. LD, ZZ, BZ, SL, YH, YL, PL, HZ, ZT, and XM: writing—review and editing. All authors contributed to the article and approved the submitted version.

## Footnotes

1
http://hannonlab.cshl.edu/fastx_toolkit/

2
https://card.mcmaster.ca
