# Supplementary material for: Research on safety and compliance of imported microbial inoculants using high-throughput sequencing
Source: Front Med (Lausanne). 2022 Sep 21;9:963988. doi: 10.3389/fmed.2022.963988 (PMC9532531; doi:10.3389/fmed.2022.963988)

**Supplementary Table 1** The primer sequences and reaction conditions for qPCR verification.

| Target | Primer Sequences | Reaction conditions |
| --- | --- | --- |
| *Bifidobacterium* | F: GAT CTG CAA GAG GAC AAA CC  R: TAC CCT GCA TTC CTT GTC GC | 30 s initial denaturation at 95 °C, 40 cycles of 5 s for denaturation at 95 °C, 31 s for annealing at 60 °C and 15 s for extension at 95 °C. |
| Denitrifying bacteria | F: TCG TCG GCA GCG TCA GAT GTG TAT AAG AGA CAG  R: GTC TCG TGG GCT CGG AGA TGT GTA TAA GAG ACA G | 3 min initial denaturation at 95 °C, 35 cycles of 10 s for denaturation at 95 °C, 30 s for annealing at 56 °C and 20 s for extension at 70 °C |
| *Saccharomyces* | F: GCA TCG ATG AAG AAC GCA GCG AAA T  R: ATT GCT CAA CAC CAA ACC CG | 5 min initial denaturation at 94 °C, 40 cycles of 10 s for denaturation at 94 °C, 20 s for annealing at 60 °C and 30 s for extension at 72 °C |
| Photosynthetic bacteria | F: TGG TYT GAG AGG ATG RYC A  R: CGA ATT TCA CCT CTA CAC TCG | 1 min initial denaturation at 95 °C, 40 cycles of 10 s for denaturation at 95 °C, 20 s for annealing at 60 °C and 31 s for extension at 72 °C |

**Supplementary Table 2** Data quality control statistics of raw data.

| Sample number | Raw data  (Mbp) | Valid data  (Mbp) | Q20  (%) | Q30  (%) | GC content  (%) | Effective rate  (%) |
| --- | --- | --- | --- | --- | --- | --- |
| Y-1 | 2036.81 | 1967.97 | 97.30 | 93.23 | 39.52 | 96.62 |
| Y-2 | 1571.59 | 1498.04 | 96.65 | 91.84 | 45.86 | 95.32 |
| Y-3 | 1775.42 | 1710.26 | 97.16 | 92.68 | 44.46 | 96.33 |
| Y-4 | 1687.67 | 1632.48 | 97.31 | 93.24 | 39.97 | 96.73 |

**Supplementary Table 3** Drug resistance genes.

| Gene types | Sample number | | | |
| --- | --- | --- | --- | --- |
|  | Y-1 | Y-2 | Y-3 | Y-4 |
| *AAC(6')-Ii* | 1 |  |  | 1 |
| *aadK* | 1 |  | 1 | 1 |
| *ANT(4')-Ib* | 1 |  |  | 1 |
| *Bacillus subtilis mprF* | 1 | 1 | 2 | 2 |
| *BcI* |  |  |  | 1 |
| *bcrA* | 1 | 1 | 3 | 1 |
| *bcrB* | 1 |  | 1 | 1 |
| *bcrC* | 1 |  | 1 | 1 |
| *Bla1* | 1 |  |  | 1 |
| *blt* | 1 | 1 | 2 | 2 |
| *bmr* | 1 |  | 1 | 2 |
| *cfr(B)* |  |  | 1 |  |
| *clbA* |  | 1 | 1 | 1 |
| *CRP* |  |  | 1 |  |
| *efmA* | 1 |  |  | 1 |
| *efrA* | 1 |  |  | 1 |
| *efrB* | 1 |  |  | 1 |
| *ErmD* | 1 |  | 1 | 1 |
| *FosM1* | 1 |  | 2 | 2 |
| *FosM2* |  | 1 | 1 |  |
| *FosM3* | 1 |  |  | 1 |
| *H-NS* | 1 |  |  |  |
| *lmrB* | 1 | 1 | 1 | 1 |
| *lsaA* | 1 |  |  | 1 |
| *lsaB* |  |  | 1 |  |
| *mphK* | 1 |  |  | 1 |
| *msrC* | 1 |  |  | 1 |
| *QnrD1* | 1 |  | 1 |  |
| *rphB* | 2 | 1 | 2 | 2 |
| *tet(L)* | 1 | 1 | 1 | 1 |
| *tet(U)* |  | 1 | 1 |  |
| *tmrB* | 1 | 1 | 2 | 1 |
| *vanR gene in vanF cluster* |  |  | 1 |  |
| *vanR gene in vanM cluster* |  |  | 1 |  |
| *vanS gene in vanF cluster* |  |  | 1 |  |
| *vanS gene in vanM cluster* |  |  | 1 | 1 |
| *vanY gene in vanF cluster* |  |  | 1 |  |
| *vanZ gene in vanF cluster* |  |  | 1 |  |
| *VatI* |  |  | 1 |  |
| *vmlR* | 1 | 1 | 2 | 2 |
| *ykkC* | 2 | 1 | 3 | 3 |
| *ykkD* | 2 | 1 | 3 | 3 |
| total | 31 | 13 | 42 | 39 |

**Supplementary Figure 1** Detection results of qPCR. *Bifidobacterium* **(A)**, *Denitrifying bacteria* **(B)**, *Saccharomyces* **(C)** and *Photosynthetic bacteria* **(D)**.


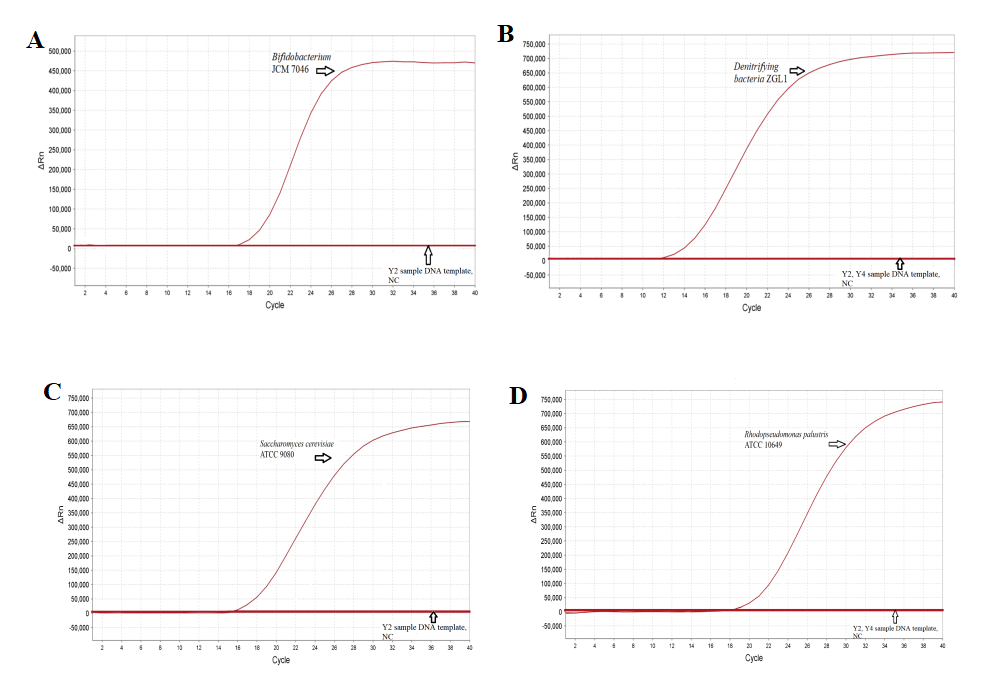

Supplement: Supplementary file 1 [file Data_Sheet_1.docx]
